# The association between new onset atrial fibrillation and incident cancer—A nationwide cohort study

**DOI:** 10.1371/journal.pone.0199901

**Published:** 2018-06-28

**Authors:** Chi-Sheng Hung, Chia-Hsuin Chang, Jou-Wei Lin, Yi-Lwun Ho, Ming-Fong Chen

**Affiliations:** 1 Telehealth Center, National Taiwan University Hospital, Taipei, Taiwan; 2 Department of Internal Medicine, National Taiwan University Hospital, Taipei, Taiwan; 3 Cardiovascular Center, National Taiwan University Hospital, Yun-Lin Branch, Douliu City, Yun-Lin County, Taiwan; 4 Clinical Outcome Research and Training Center, China Medical University Hospital, College of Medicine, China Medical University, Taichung, Taiwan; 5 Cardiovascular Center, China Medical University Hospital, College of Medicine, China Medical University, Taichung, Taiwan; University of Palermo, ITALY

## Abstract

A recent analysis showed an association with new onset atrial fibrillation (NOAF) and incident cancer among women. We aimed to examine the risk of cancer among patients with NOAF in general population. A retrospective cohort of 5130 patients with NOAF was identified from a random sample of one million subjects between 2005 and 2010 from Taiwan National Health Insurance Research Database. The standard incidence ratio of incident cancer and hazard ratios were calculated by modeling cumulative incidence with competing risk of death. During a mean follow-up duration of 3.4 years, 330 patients developed cancer. The standard incidence ratio of all malignancies was 1.41 (95% confidence interval 1.26–1.57), suggesting a 41% increase in cancer risk compared with the general population. The risk of cancer was higher among men or the elderly with NOAF after adjusting for confounding factors and after considering the competing risk of death. The risk of cancer was not associated with CHA2DS2–VASc score (p = 0.32) among patients with NOAF. In conclusion, patients with NOAF were associated with a higher risk of cancer. Within this group, the risk of ischemic stroke (in terms of CHADS2-VASc score) did not reflect the risk of incident cancer.

## Introduction

Atrial fibrillation (AF) is one of the most common arrhythmias and is associated with many cardiovascular complications, especially ischemic stroke. AF is frequently induced either by cardiovascular disease[1, 2] or by non-cardiovascular diseases.[3, 4]

According to the latest studies, there is also an association between AF and new onset malignancies.[5, 6] In a case-control study, the diagnosis of AF was more frequent among patients with newly diagnosed colorectal and breast cancer.[5] In a large observational study, among 1467 healthy women, newly diagnosed AF was associated with incident malignancy after a median follow-up of 19.1 years.[6] In two recent studies in a population of AF patients taking anticoagulants, malignancy was the major non-cardiovascular cause of death (10.3%–13.9%) and was even higher than deaths caused by ischemic stroke (5.1%–7.0%).[7, 8] Therefore, to further improve the outcome of AF, it is important to clarify the association between these two diseases.

To explore the relationship between AF and malignancy, we designed this observational study using data from a random sample of one million subjects from the Taiwan National Health Insurance Research Database (NHIRD). We aimed to examine 1) whether the risk of incident cancer was higher among patients with newly diagnosed AF and 2) whether the CHADS2-VASc score was associated with the risk of incident cancer.

## Materials and methods

### Data sources

The protocol of this study was approved by the Institutional Review Board of the National Taiwan University Hospital (NTUH IRB 201704010W) to analyze the anonymized data, and complied with the Helsinki Declaration. Permission to waive informed consent has been obtained. All methods were performed in accordance with the relevant guidelines and regulations. The NHIRD includes complete demographic data, outpatient visits, hospital admissions, and pharmacies dispensing claims for around 98.4% of the country’s population. The Longitudinal Health Insurance Database 2005, a subset of NHIRD, comprises one million individuals randomly selected from the NHIRD in 2005. Then the original claim data of these one million individuals from 2000 to 2011were temporally linked to make a large longitudinal cohort.[9] The source population used in this study included all individuals from the Longitudinal Health Insurance Database 2005 who were at least 20 years of age on January 1, 2001.

### Study population

From the source population, we identified individuals aged 20 years and above who developed new-onset AF. New-onset AF (NOAF) was defined as a new AF (ICD-9CM: 427.31) diagnosis at least twice in the records of outpatient clinics within one year or at least once during hospitalization. The index date of AF onset was defined as the first date AF was diagnosed. Patients aged less than 20 years old, who had an AF diagnosis before the index date or who had a cancer diagnosis (ICD-9-CM: 140–208) were excluded.

### Outcome definition

The primary outcome was incident malignancy (ICD-9-CM: 140–208). The secondary outcomes were different pre-specified types of malignancies: lung cancer (ICD-9: 162), colon cancer (ICD-9: 153–154), liver cancer (ICD-9: 155), and breast cancer (ICD-9: 174).

### Statistical analysis

Categorical data were reported as numbers and percentages. The continuous variables were presented as mean ± SD. The categorical variables were compared with Pearson’s Chi-square test, while continuous variables were compared with Student`s t-test or Wilcoxon signed-rank test as appropriate. Standardized incidence ratio (SIR) was measured by comparing observed to expected numbers of incident cancer in the general Taiwanese population. The expected numbers of incident cancer were derived from the incidence rates stratified by age (5 years as a group) and sex. The incident cancer was identified from the National Health insurance registry files if a patient received a “certification of cancer,” which was a government issued document proving the diagnosis of cancer. To test if CHA2DS2-VASc score was able to predict future cancer risk, we modeled the cumulative incidence for incident cancer using the subdistribution hazards models proposed by Fine and Gray[10].

A two-tailed p-value < 0.05 was considered statistically significant for all analyses. Statistical analyses were performed using SAS version 9.3 (SAS Institute, Cary, NC).

## Results

### Baseline characteristics

**Fig 1:**
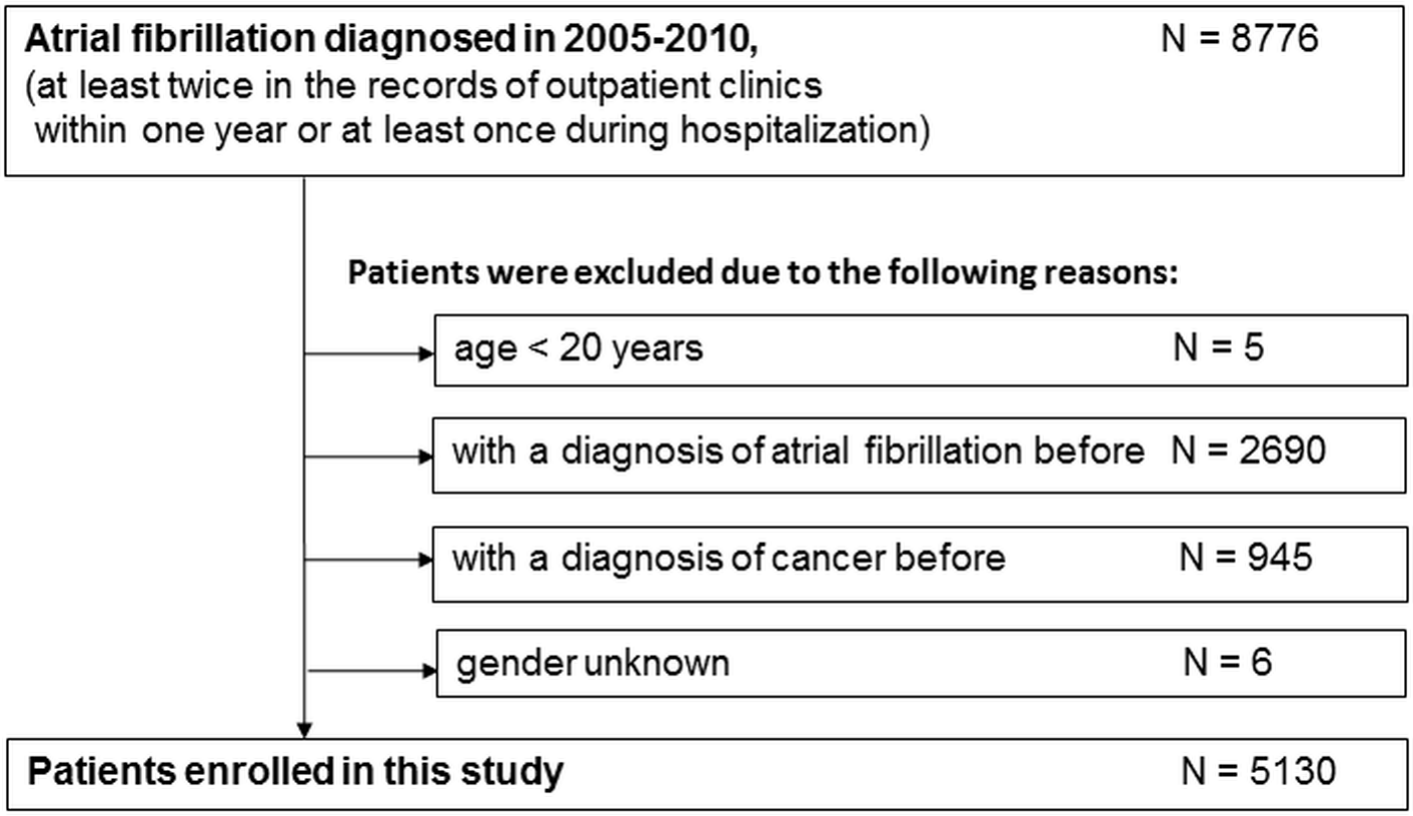
Diagram of patient flow.

There were 8776 patients with AF between 2005 and 2010 in this cohort. After excluding patients aged below 20 years or who had an AF or malignancy diagnosis before 2005, 5130 patients were included in our study (Fig 1). Among these patients, 2749 (53.6%) were men; the mean age was 71.4 ±13.6 years old, and the median CHA2DS2-VASc score was 4 (interquartile range [IQR] 3–6). The baseline characteristics are shown in Table 1.

**Table 1:** Baseline characteristics.

			CHA2DS2-VASc scores				
Variable	Category	Total	0–2	3–4	5–6	7–9	p-value
Patient number		5130 (100.0)	1270 (100.0)	1716 (100.0)	1436 (100.0)	708 (100.0)	—
**Demographic data**							
Age in years	Mean (SD)	71.4 (13.6)	58.0 (13.2)	72.0 (11.5)	78.0 (8.9)	80.9 (6.7)	< .0001
Age	<65	1395 (27.2)	901 (70.9)	403 (23.5)	82 (5.7)	9 (1.3)	<0.001
	65–74	1268 (24.7)	274 (21.6)	573 (33.4)	340 (23.7)	81 (11.4)	
	75+	2467 (48.1)	95 (7.5)	740 (43.1)	1014 (70.6)	618 (87.3)	
Gender	Male	2749 (53.6)	994 (78.3)	939 (54.7)	620 (43.2)	196 (27.7)	<0.001
Follow-up time in years	Mean (SD)	3.4 (2.0)	3.7 (1.9)	3.5 (1.9)	3.2 (2.0)	2.8 (2.0)	
Cancer observed		330 (6.4)	64 (5.0)	129 (7.5)	98 (6.8)	39 (5.5)	
Death, no cancer observed		1182 (23.0)	127 (10.0)	330 (19.2)	423 (29.5)	302 (42.7)	
**Comorbidities**							
Hypertension		3477 (67.8)	366 (28.8)	1171 (68.2)	1258 (87.6)	682 (96.3)	<0.001
Diabetes		2981 (58.1)	408 (32.1)	927 (54.0)	1027 (71.5)	619 (87.4)	<0.001
Ischemic heart disease		2016 (39.3)	142 (11.2)	566 (33.0)	776 (54.0)	532 (75.1)	<0.001
Myocardial infarction		210 (4.1)	11 (0.9)	60 (3.5)	74 (5.2)	65 (9.2)	<0.001
Chronic renal failure		391 (7.6)	36 (2.8)	115 (6.7)	140 (9.7)	100 (14.1)	<0.001
Chronic liver disease		605 (11.8)	143 (11.3)	218 (12.7)	156 (10.9)	88 (12.4)	0.364
Chronic lung disease		1813 (35.3)	249 (19.6)	589 (34.3)	607 (42.3)	368 (52.0)	<0.001
Depression		305 (5.9)	41 (3.2)	80 (4.7)	105 (7.3)	79 (11.2)	<0.001
Congestive heart failure		1326 (25.8)	56 (4.4)	302 (17.6)	546 (38.0)	422 (59.6)	<0.001
Vascular disease		2162 (42.1)	150 (11.8)	606 (35.3)	848 (59.1)	558 (78.8)	<0.001
Stroke/TIA		1272 (24.8)	8 (0.6)	163 (9.5)	504 (35.1)	597 (84.3)	<0.001
Charlson’s comorbidity index	Mean (SD)	2.0 (1.7)	0.8 (1.0)	1.6 (1.3)	2.6 (1.5)	3.9 (1.6)	< .0001
**Medications**							
Oral anti-diabetic agents		1084 (21.1)	86 (6.8)	306 (17.8)	435 (30.3)	257 (36.3)	<0.001
Fast-acting insulin		491 (9.6)	31 (2.4)	89 (5.2)	203 (14.1)	168 (23.7)	<0.001
ACE inhibitors		1642 (32.0)	159 (12.5)	523 (30.5)	590 (41.1)	370 (52.3)	<0.001
Angiotensin receptor blockers		1450 (28.3)	139 (10.9)	437 (25.5)	527 (36.7)	347 (49.0)	<0.001
Beta-blockers		2679 (52.2)	463 (36.5)	893 (52.0)	849 (59.1)	474 (66.9)	<0.001
Calcium channel blockers		3154 (61.5)	387 (30.5)	1066 (62.1)	1108 (77.2)	593 (83.8)	<0.001
Diuretics		2667 (52.0)	272 (21.4)	862 (50.2)	976 (68.0)	557 (78.7)	<0.001
Statins		864 (16.8)	101 (8.0)	271 (15.8)	304 (21.2)	188 (26.6)	<0.001
Fibrates		303 (5.9)	44 (3.5)	104 (6.1)	106 (7.4)	49 (6.9)	0.000
Digitalis glycoside		1115 (21.7)	135 (10.6)	329 (19.2)	414 (28.8)	237 (33.5)	<0.001
Antiarrhythmics class I and III		942 (18.4)	147 (11.6)	284 (16.6)	318 (22.1)	193 (27.3)	<0.001

### Outcome

During a mean follow-up duration of 3.4 years, 330 patients (6.4%) developed cancer. During the same period, 1182 patients (23%) died without cancer been diagnosed. The incidence of all cancer after NOAF was 19.77 per 1000 person-years (13.98 in female and 24.86 in male) (Table 2). Among the whole study population, the most common types of cancer were lung (n = 58, incidence: 3.48 per 1000 person-years), colon (n = 55, incidence: 3.3 per 1000 person-years) and liver (n = 35, incidence: 2.1 per 1000 person-years) (Table 2).

**Table 2:** Crude incidence rates of outcomes.

						CHA2DS2-VASc scores															
		Total				0–2				3–4				5–6				7–9			
Subgroup		N	py	E	IR*	N	py	E	IR*	N	py	E	IR*	N	py	E	IR*	N	py	E	IR*
**All cancers**																					
All patients		5130	16688	330	19.77	1270	4591	64	13.94	1716	5810	129	22.2	1436	4384	98	22.35	708	1903	39	20.5
Gender	Female	2381	7799	109	13.98	276	1046	9	8.61	777	2761	36	13.04	816	2553	45	17.63	512	1439	19	13.2
	Male	2749	8889	221	24.86	994	3545	55	15.51	939	3049	93	30.5	620	1831	53	28.94	196	463	20	43.16
Age	<65	1395	5257	55	10.46	901	3381	35	10.35	403	1562	12	7.68	82	293	7	23.9	9	22	1	46.32
	65–74	1268	4330	109	25.17	274	925	22	23.78	573	1970	55	27.91	340	1181	26	22.02	81	254	6	23.65
	75+	2467	7101	166	23.38	95	285	7	24.56	740	2278	62	27.22	1014	2910	65	22.33	618	1628	32	19.66
**By type of cancer**																					
Breast cancer		2381	7799	12	1.54	276	1046	2	1.91	777	2761	4	1.45	816	2553	4	1.57	512	1439	2	1.39
Colon cancer		5130	16688	55	3.3	1270	4591	10	2.18	1716	5810	25	4.3	1436	4384	10	2.28	708	1903	10	5.26
Liver cancer		5130	16688	35	2.1	1270	4591	6	1.31	1716	5810	15	2.58	1436	4384	9	2.05	708	1903	5	2.63
Lung cancer		5130	16688	58	3.48	1270	4591	15	3.27	1716	5810	21	3.61	1436	4384	19	4.33	708	1903	3	1.58

* per 1000 person-years

Abbreviations: N, patient no.; py, person year; E, event no.; IR, incidence rate

**Fig 2:**
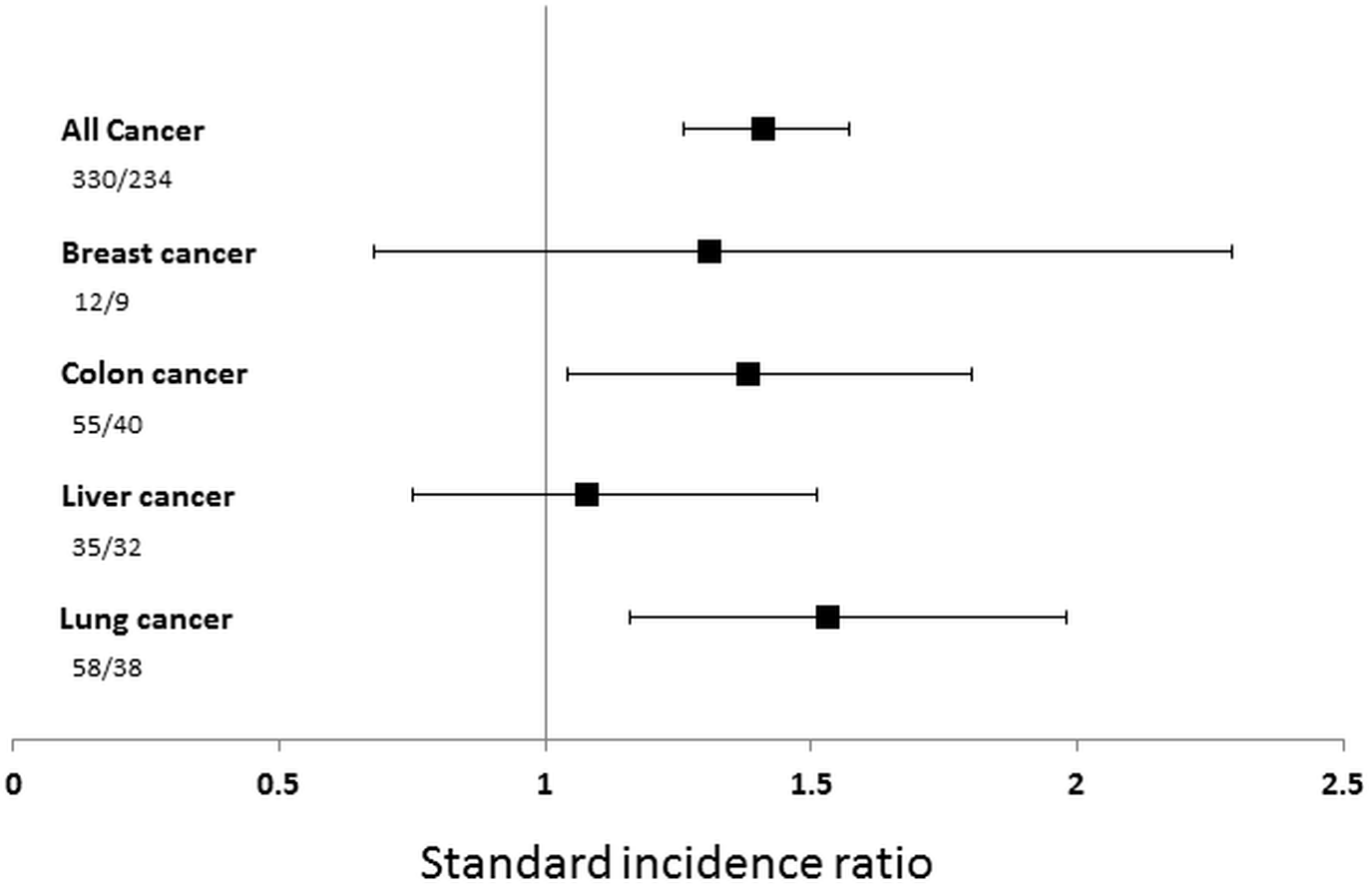
The standardized incidence ratio. The standardized incidence ratio and 95% confidence interval of all cancers and different types of cancer compared with the general population. Numbers shown are observed number of cancer cases/expected number of cancer cases.

Compared to the general population, the standard incidence ratio (SIR) of all malignancies was 1.41 (95% confidence interval 1.26–1.57), suggesting a 41% increase in cancer risk compared with the general population. The SIR of lung and colon cancer was significantly higher than expected (1.53 [95%CI 1.16–1.98] and 1.38 [95%CI 1.04–1.80], respectively). However, there was no statistically significant increase in the relative risk of liver or breast cancer (Fig 2).

The SIR for all cancer was 1.23 (95%CI 1.01–1.48) in women and 1.52 (1.33–1.83) in men. The SIR for all cancer was 1.71 (95%CI 1.29–2.23) in patients aged under 65, 1.81 (95%CI 1.48–2.18) in patients aged between 65 and 74, and 1.17 (95%CI 1.00–1.36) in patients aged above 75 (Fig 2). Because the percentage of patients in this cohort that died was higher than that of patients diagnosed with new cancer, we modeled cancer incidence considering a competing risk of death. The risk of all cancer was higher among men than women (hazard ratio 2.00, 95%CI 1.57–2.56) and within the elderly patient group compared with youngest group after adjusting for confounding factors and considering the competing risk of death (Table 3). The risk for all cancer, however, was not associated with CHA2DS2–VASc score (p = 0.321). Similarly, there was no positive relationship between the risk of ischemic stroke and incident cancer among all subtypes of cancer.

**Table 3:** Relative risk of cancer incidence by modeling the cumulative incidence while considering the competing risk of death (Fine and Gray’s subdistribution method).

Covariate	All cancer			Lung cancer			Colon cancer			Liver Cancer			Breast Cancer		
	HR	95% CI	p-value	HR	95% CI	p-value	HR	95% CI	p-value	HR	95% CI	p-value	HR	95% CI	p-value
CHA2DS2-VASc scores			0.3208			0.4485			0.0993			0.7814			0.8734
0–2	ref.			ref.			ref.			ref.			ref.		
3–4	1.34	0.97–1.85		0.80	0.38–1.71		1.95	0.84–4.54		1.40	0.56–3.50		1.03	0.20–5.27	
5–6	1.24	0.85–1.81		0.88	0.40–1.97		1.00	0.32–3.15		1.10	0.41–2.97		1.70	0.24–11.99	
7–9	1.14	0.70–1.84		0.34	0.09–1.32		2.33	0.69–7.89		1.69	0.52–5.48		2.19	0.25–19.24	
Gender			<0.0001			0.0008			0.0351			0.0009			
Female	ref.			ref.			ref.			ref.					
Male	2.00	1.57–2.56		2.71	1.51–4.84		1.92	1.05–3.51		4.27	1.82–10.03				
Age			<0.0001												
<65	ref.			ref.		0.0123	ref.		0.7065	ref.		0.0043	ref.		0.0719
65–74	2.24	1.58–3.16		3.80	1.56–9.25		1.35	0.60–3.05		9.39	2.26–39.02		0.94	0.24–3.64	
> = 75	1.72	1.19–2.48		2.85	1.07–7.58		1.10	0.44–2.78		4.88	1.20–19.81		0.12	0.02–1.03	

Cancer incidence was generally the highest in the 65–74 years group, followed by the >75 years group. Further, except for women with new onset AF, increasing age was not associated with a higher risk of incident breast cancer.

## Discussion

The main findings of this large population-based cohort study are as follows: (1) patients with NOAF were associated with higher incidence of all cancer compared with the general population after adjusting for age and sex; the association was significant in both men and women and in all age subgroups (2) the association was significant in lung and colorectal cancer, but not in breast or liver cancer; and (3) the CHADS2-VASc score was not associated with risk of incident cancer in patients with NOAF. Our results agreed with several previous reports on the increased association between NOAF and incident cancer.[6, 11, 12] The thromboembolic risk of AF, as indicated by the CHADS2-VASc score, was not related to the incidence of new cancer.

Recently, two large cohort studies reported a positive association between NOAF and incident cancer [6, 11]. In the Danish nationwide retrospective cohort study, the SIR of incident cancer was 5.11 (4.99–5.24) among patients with NOAF within 3 months after the diagnosis of AF. However, the SIR after 3 months of AF diagnosis was only slightly higher than expected (1.38 [1.32–1.45] at 4–6mo). The authors concluded that occult cancer was likely to be present at the time of AF diagnosis.[11] In the large Women’s Health Study among 34691 women in US with a median follow-up of 19.1 years, NOAF was significantly associated with higher incident cancer in multivariable-adjusted Cox-models (HR 1.48 [95%CI 1.25–1.75]). Similar to the Danish study, the risk was significantly higher within the first 3 months after AF diagnosis, but not thereafter.[6] In a letter to the Women’s Health Study, Kim et al. found a similar association between NOAF and incident cancer in a larger cohort.[12] Taken together, these data suggest that the diagnosis of new cancer is more frequently made among patients with NOAF, especially within the 3-month period after the diagnosis of AF.

The mechanisms of this association, however, are not yet clear. Cancer has been shown to predispose the development of AF, either post-operatively or in non-surgical patients.[13, 14] Systemic inflammation is one of the possible mechanisms linking these two conditions. In a case-control study, cancer was associated with higher CRP level and higher risk of AF.[15] It is possible that occult cancer increases systemic inflammation and then leads to the development of NOAF, which is detected earlier than cancer itself. Furthermore, amiodarone, the drug used frequently in rhythm control for AF, has been reported to be associated with higher risk of incident cancer, although the mechanism is also lacking. [9] Other potential mechanisms linking these two conditions, including shared risk factors and detection bias, have been proposed. Shared risk factors, including advanced age, obesity, smoking and alcohol, may predispose patients to both conditions. Detection bias is also possible in this association. Patients with NOAF may undertake more examinations than patients without AF, which leads to a higher probability of detecting existing, but undiagnosed, cancer. However, detection bias cannot explain why the association is only observed in some, but not all, subtypes of cancer.

Cancer is commonly associated with systemic inflammation and thromboembolism. According to a recent randomized controlled trial, 3.9% of patients with a new diagnosis of initial unprovoked venous thromboembolism (VTE) were later found to have a new diagnosis of occult cancer within a one-year follow-up period.[16] A risk score including age, gender, chronic lung disease, platelet count, and surgery was developed to predict the presence of occult cancer in patients with VTE, but with only modest accuracy.[17] Our data showed that 6.4% of patients with new onset AF would be later diagnosed with cancer. The incidence rate may be comparable with that in VTE. However, the association and diagnosis for occult cancer in patients with new onset AF may have been largely overlooked. Our work is a pilot investigation to test if a conventional thromboembolism prediction score could be applied to predict incident cancer. Our results suggest the need for a predictive model to fulfill this need.

Among the different subtypes of cancer, the association between NOAF and colorectal cancer has been consistently reported in our study and in previous reports.[6, 11]. In the Women’s Health Study, the adjusted HR for colon cancer was 2.11, while the standardized incidence rate of colon cancer was 1.13 in a Danish cohort and 1.38 in our report. In a case-control study, the presence of AF was associated with a higher risk of colon cancer (OR 11.8). Apart from shared risk factors, systemic inflammatory process, and detection bias, anticoagulation-related bleeding complications might unmask pre-existing colon cancer among patients with NOAF. Gastrointestinal (GI) bleeding is a common complication after initiating anticoagulants. This anticoagulation-related bleeding complication can increase the extent of GI cancer detected in patients with NOAF using warfarin or novel oral anticoagulation.[18, 19] The incidence of colon cancer was 0.5% compared with 0.05% in cancers other than GI origin among patients with AF treated by dabigatran in the RE-LY trial.[19] Hence, the authors coined the term “anticoagulation GI stress test”. Although evidence is still lacking, it seems prudent to perform a screen test for stool occult blood in patients with NOAF within a few months after starting anticoagulation. Moreover, anticoagulation used in AF patients with hematuria can be associated with a higher prevalence of genitourinary cancer.[20] The association between NOAF and genitourinary cancer warrants further confirmation using a large population study.

Recently, the hypothesis of shared genetic risk factor has been proposed to explain the relationship between AF and cancer.[21] Tumor suppressor genes play crucial role to protect cell from malignant transformation. The loss or reduction of tumor suppressor gene expression relieves the negative regulation of cell proliferation, and then, in combination with other genetic changes, leads to malignant transformation. Recent studies have identified two tumor suppressor genes, Zinc finger homeobox protein-3 (ZFHX3) and esophageal cancer related gene-4 (ECRG4), are closely related to the pathogenesis of AF.[21] Alteration in the expression of ZFHX3 has been reported in colon cancer[22], which also links to AF in our results. Other mutations in connexins and p53 have also been shown to be associated with the development of both cancer and AF. Although to what extend do these mutations contribute to the association between AF and cancer is not yet clear, these observations do suggest a shared molecular mechanism is possible.

The strengths of this study include using a database from a nationwide, single-payer health care system. We incorporate the CHA2DS2-VASc score in our analyses. The CHA2DS2-VASc score can be associated with the incidence of NOFA in a large population.[23] The higher score is also associated with a higher risk of ischemic stroke. Our analyses revealed that the risks for ischemic stroke and cancer were not parallel. We also considered the competing risk of death in our model. There are several limitations in our study. First, because the study was derived from Taiwan’s nationwide health care system, the majority of participants were Asians. Therefore, we cannot generalize results to other races. Second, because the diagnosis of AF was based on the ICD-9 code, we cannot generalize to patients with asymptomatic or paroxysmal AF, which would be under-estimated by the diagnosis code.

## Conclusion

The results showed that NOAF was associated with the risk of incident cancer, especially lung and colon cancer. The CHADS2-VASc score was not associated with the risk of incident cancer in patients with NOAF.
